# Potential Role of *ABCF2* Gene in Pudendal Nerve Neuropathy and Interstitial Cystitis

**DOI:** 10.3390/genes16030281

**Published:** 2025-02-26

**Authors:** Antonino Musumeci, Mirella Vinci, Simone Treccarichi, Alda Ragalmuto, Giuseppe Bruno, Giordana Tinniriello, Jessica Farina, Concetta Federico, Salvatore Saccone, Francesco Calì, Daniele Porru

**Affiliations:** 1 Oasi Research Institute-IRCCS, 94018 Troina, Italy; amusumeci@oasi.en.it (A.M.); mvinci@oasi.en.it (M.V.); streccarichi@oasi.en.it (S.T.); aragalmuto@oasi.en.it (A.R.); 2 Department of Medical and Surgical Sciences and Advanced Technologies “G.F. Ingrassia”, Anatomic Pathology, University of Catania, 95131 Catania, Italy; g.bruno2658@gmail.com (G.B.); giordanatinnirello@yahoo.it (G.T.); jessicafarina2693@gmail.com (J.F.); 3 Department Biological, Geological and Environmental Sciences, University of Catania, Via Androne 81, 95124 Catania, Italy; concetta.federico@unict.it (C.F.); salvatore.saccone@unict.it (S.S.); 4 Urology Department, Fondazione IRCCS, San Matteo Hospital, 27100 Pavia, Italy; danieleporru@tin.it

**Keywords:** pudendal nerve neuropathy, interstitial cystitis, urothelial destruction, ATP-binding cassette sub-family F member 2, ATP metabolism, whole exome sequencing, Sanger sequencing, genetic variant

## Abstract

Background/Objectives: Symptoms of pudendal nerve neuropathy may overlap with various symptoms of interstitial cystitis (IC). As documented, there is a well-established correlation between the genes involved in ATP metabolism, neuropathy, and IC. ATP-binding cassette (ABC) transporters genes, in fact, are vital for ATP signaling. This study aims to associate the *ABCF2* gene with a suspected pudendal nerve neuropathy and IC. Methods: Histological analysis was conducted for diagnosing IC while the genetic variant was identified by whole exome sequencing (WES) Trio and confirmed through Sanger. Results: We report a patient with IC, confirmed by histological examination, presenting with a suspected bladder and pudendal nerve neuropathy, though not analytically confirmed. Histological analysis revealed urothelial detachment caused by a dense subepithelial lymphocytic infiltrate, predominantly composed of mast cells, which serve as key diagnostic markers for interstitial cystitis (IC). WES analysis identified the heterozygous genetic variant c.1253T>G p.Phe418Cys within *ABCF2* gene, precisely in its functional domain which actively operates in the hydrolysis of ATP energizing various biological systems. As reported, this gene displays high expression patterns in bladder tissue. The variant, absent in the healthy brother, was inherited from the father which presents mosaicism. The in silico prediction analyses classified this variant as pathogenic, identifying potential alterations in the protein structure. Conclusions: Although the precise role of *ABCF2* should be supported by further studies, we hypothesize that its disruption might impair ATP metabolism, likely altering the nociceptive response and leading to the patient’s neuropathy. Further analyses are imperative to validate this research, for laying the groundwork for a specific therapy targeting the genetic dysregulation involved in this condition.

## 1. Introduction

Pudendal neuralgia, a chronic condition causing neuropathic pelvic pain, is frequently misdiagnosed and inappropriately treated. The Nantes criteria assist in diagnosing pudendal neuralgia caused by pudendal nerve entrapment [1,2]. The pudendal nerve supplies areas of the pelvis, and its entrapment or damage can cause pelvic pain syndromes, including bladder pain syndrome/interstitial cystitis (BPS/IC), characterized by pressure and discomfort. As documented, there exists an established relationship between the pudendal canal and nerve and IC [3,4,5]. This debilitating heterogeneous condition with unknown causes is characterized by persistent pelvic pain and lower urinary tract symptoms. It encompasses a broad array of phenotypes characterized by different possible etiologies. Despite BPS/IC is more common in women, it may affect both sexes presenting at various ages, often associated with chronic pain, depression, anxiety, and other mental health issues. Recent evidence suggests that IC/BPS should be thoroughly distinguished based on the presence or absence of Hunner lesions, rather than by symptoms alone. In fact, these represent different inflammatory conditions [6]. Within this context, the Hunner lesion subtype is an inflammatory disease with bladder-specific etiology, marked by epithelial damage, heightened immune responses, and clonal expansion of B cells, possibly linked to infection. Evidence indicates that IC/BPS with Hunner lesions is a distinct inflammatory bladder condition, while other IC/BPS forms lack bladder pathology, involving neural dysfunction and central sensitization with possible urothelial issues and broader somatic or psychological symptoms [6].

Conversely, the non-Hunner lesion subtype is a non-inflammatory disorder, probably related to urothelial dysfunction and neurophysiological issues, often accompanied by somatic and psychological symptoms leading to central nervous sensitization [6]. As previously reported, Hunner lesions are present in 5–57% of patients exhibiting IC/BPS [7]. Despite a specific efficient therapy for IC/BPS not being assigned yet, according to international guidelines neuromodulation is recommended as a fourth-line treatment for BPS/IC patients who do not respond to behavioral therapies, oral medications, intravesical treatments, or hydrodistension [8].

Several authors have emphasized the potential genetic cause attributed to this syndrome. Gene expression studies have identified differential expression levels of various genes involved in ATP metabolism. Specifically, several studies have shown how uncontrolled ATP release within urothelial cells results from increased intracellular calcium concentrations. The genes found to exhibit significantly increased expression levels are *TRPA1*, *TNFRSF17*, *S1PR4*, *HIF1A*, *CXCL8*, *CXCL1*, *IL6*, and *P2RX7*. Several of them have been considered as potential biomarkers, although confirmation remains uncertain [9,10,11,12,13,14].

Within this context, ABC transporters, known for their role in cellular efflux transport, and purinergic receptors, involved in ATP metabolism and signaling, are both implicated in neuropathic conditions such as neuropathy and bladder dysfunction [15,16]. These molecular pathways play crucial roles in modulating inflammatory responses, with aberrations potentially contributing to the pathogenesis of diverse inflammatory diseases, such as the rare IC [9]. In fact, dysregulation of the ATP metabolism has been previously associated with IC [17,18].

ATP-binding cassette (ABC) transporters are integral membrane proteins that facilitate ATP-dependent translocation of various substrates across cell membranes, crucial for nutrient uptake, drug resistance, and detoxification [19]. Their function involves ATP-binding and hydrolysis, driving conformational changes in transmembrane domains to control substrate transport [20]. Eukaryotic ABC proteins are classified into seven subfamilies (ABCA–G) based on sequence homology, transmembrane domain (TMD) architecture, and auxiliary domains. Among these, ABCF transporters exhibit structural and functional diversity specific to their cellular roles. Unlike other subfamilies, ABCE and ABCF lack TMDs and are involved in non-transport-related processes [19]. ABCF genes are upregulated by tumor necrosis factor-α, indicating potential involvement in inflammatory pathways, although no diseases have been directly linked to ABCE or ABCF genes [21]. Among ABCF genes, *ABCF2* is notable as a potential cancer biomarker. It is a valuable prognostic indicator for uterine cervical cancer and is expressed in various bladder tissues. Elevated *ABCF2* DNA copy number and mRNA levels are observed in clear cell ovarian adenocarcinomas compared to serous ovarian cystadenocarcinomas, correlating with chemotherapy response and prognosis in clear cell adenocarcinoma of the ovary [22].

The current work aims to establish a potential correlation between a genetic variant within *ABCF2*, identified by whole exome sequencing (WES), and a suspected pudendal nerve neuropathy in a patient diagnosed with IC.

## 2. Materials and Methods

### 2.1. Histological Analysis

Interstitial cystitis (IC) was diagnosed according to the National Institutes of Health (NIH) criteria, which focus on the presence of irritative voiding symptoms without other identifiable pathology [23]. The European Society for the Study of Bladder Pain Syndrome (ESSIC) recommends cystoscopic investigation to identify increased mast cells in the detrusor muscle for diagnosing bladder pain syndrome/interstitial cystitis (BPS/IC) [24]. A cystoscopic examination was performed under anesthesia, and a bladder biopsy sample was taken using closed cup forceps through a flexible or rigid cystoscope after hydrodistension. The severity of the cystoscopic findings correlated with histological evidence of inflammation in the patient with IC and suspected pudendal nerve neuropathy was not analytically confirmed. Bladder tissues were fixed in 10% neutral buffered formalin and embedded in paraffin. For hematoxylin-eosin staining and immunohistochemical study, the embedded bladder tissues were sectioned into 4 µm thick slices, deparaffinized, treated with 0.3% hydrogen peroxide, and incubated as previously described [25]. CD117 immunohistochemistry was used to evaluate the predominance of mast cells in the inflammatory infiltrate in the lamina propria. The role and properties of these cells in the muscularis mucosae and in the detrusor are still being discussed and remain controversial.

### 2.2. Next Generation Sequency (NGS) Analysis

Written informed consent was obtained from the patient, the brother and both the parents. Genomic DNA was extracted from peripheral blood leukocytes retrieved from the patient and the healthy parents. DNA extraction was performed according to a previously described protocol [26]. Library preparation (TRIOS) and exome enrichment were conducted using the Agilent SureSelect V7 Kit (Santa Clara, CA, USA) in alignment to the manufacturer’s instructions. A sequencing run was performed on an Illumina HiSeq 3000 Instrument (San Diego, CA, USA). This approach achieved 97% of regions covered at least 20x. The identified genetic variants were filtered according to the following criteria: (i) recessive/de novo/X-linked pattern of inheritance, (ii) allele frequencies (minor allele frequency, MAF) < 1% using as reference the following genomic datasets: 1000 Genomes, ESP6500, ExAC, GnomAD. Integrated Genomics Viewer (IGV) was employed to depict the DNA sequence. For the variant confirmation, conventional Sanger sequencing was performed using the BigDye Terminator v1.1 Cycle Sequencing Kit (Life Technologies, Carlsbad, CA, USA) with an ABI 3130 instrument (Life Technologies, Carlsbad, CA, USA) as previously described [27]. The primers were forward: 5’-ACTCAGGCCCTCCCA-3’; reverse: 5’-TGGTGACTGGGAATT–3’.

### 2.3. Data Analysis

Uniprot database (https://www.uniprot.org/) (accessed on 2 September 2024) was used to obtain the protein details related to ABCF2, regarding its functional regions and domains. The expression among the different tissue was retrieved on the specific expression databases Genotype-Tissue Expression (GTEx) (https://www.gtexportal.org/) (accessed on 2 September 2024) and The Human Protein Atlas (https://www.proteinatlas.org/) (accessed on 2 September 2024). Gene ontology (GO) terms and domain annotation were obtained from QuickGO database (https://www.ebi.ac.uk/QuickGO/) (accessed on 2 September 2024), Reactome (https://reactome.org) (accessed on 2 September 2024) and PROSITE (https://prosite.expasy.org/) (accessed on 2 September 2024). Protein structure predictions for both the wild-type and mutated ABCF2 were generated using AlphaFold prediction algorithms based on machine learning DeepMind Technologies (London, UK). In particular, the software employed was UCSF ChimeraX version 1.7 (software developed by the Resource for Biocomputing, Visualization, and Informatics at the University of California, San Francisco, with support from National Institutes of Health R01-GM129325 and the Office of Cyber Infrastructure and Computational Biology, National Institute of Allergy and Infectious Diseases) (https://www.cgl.ucsf.edu/chimerax/) (accessed on 2 September 2024). Specifically, the AlphaFold algorithm generated five models, and in accordance with its output, the “best model” was selected for this investigation. Pathogenic variants were investigated on the Human Gene Mutation Database (HGMD Professional 2023). Diverse VarAFT filters were employed on the vcf files. The observed variant was described according to the “American College of Medical Genetics” (ACMG) guidelines and was performed with VarSome. The conservations parameters used for analyzing the conservation tendency of the specific mutation region were obtained from VarSome analysis. Particularly, PhyloP100way scores are based on multiple alignments of 99 vertebrate genome sequences to the human genome. The greater score indicates a high conservation rate of the mutated site. On the other hand, PhastCons100way scores are based on 100 vertebrate genomes (including human) for identifying conserved genomic regions using multiple alignments. Its value ranges from 0 to 1 and values close to 1 indicate high conservation rate. Fitcons (FITness CONSequences of functional annotation) identifies genomic regions that are under selective pressure by integrating epigenomic signals. This method considers the genomes of 54 unrelated human individuals from the 69 sequences released by Complete Genomics. Fitcons-gm represents score from the GM12878 cell line. Its score fluctuates from 0 to 0.958517. Furthermore, the background selection (B) value ranges from 0 to 1000. It estimates the expected fraction (*1000) of neutral diversity present at a site. Values close to 0 represent near complete removal of diversity as a result of background selection and values near 1000 indicate the absence of background selection. DUET (https://biosig.lab.uq.edu.au/duet/) (accessed on 2 September 2024) and MutPred2 (http://mutpred2.mutdb.org/) algorithms (accessed on 2 September 2024) were used for assessing the destabilizing effects of the mutation.

## 3. Results

### 3.1. Clinical Report

A 33-year-old young woman was seen because in 2004 she had an appendectomy for appendicitis with peritonitis. Subsequently, she experienced recurring urinary trait infections (UTIs) from *Escherichia coli*, *Klebsiella*, every 2–3 months. In 2016, bacterial vaginosis was detected with a vaginal swab, oral, and vaginal AB, and in 2017 itching and burning of the clitoris and vaginal entry persisted. During 2009, she had vulvar warts removed. In 2016, the individual was hospitalized for cystoscopy under sedation and bladder biopsy. The observation of a significant number of mast cells in the lamina propria and detrusor layer was a finding consistent with the diagnosis of IC. Pregnancy with spontaneous birth occurred in 2017, with complete remission of urinary symptoms during pregnancy, which persisted for 4 years. In 2018, during remission an outpatient cystoscopy showed an apparently normal bladder wall and mucosa. In January 2021, due to an acute onset of clitoral pain, after urogynecological examination amitriptyline was prescribed during the 5 months of pregnancy. Spontaneous birth occurred in June 2021, which featured an exacerbation of vestibular burning and a sensation of full bladder, with more intense pain when the bladder was full. In July 2021, an outpatient cystoscopy revealed signs of diffuse mucosal hyperemia. In October 2021, a voiding diary was prepared: the voiding volume ranged from 200 mL to 600 mL, and between 3 and 5 voidings occurred during the daytime, with no voiding overnight. Rapid and regular bladder emptying, with no dyspareunia, was observed. The therapy included amitriptyline (25 mg), PEA × 2, lipoic acid tablets, hyaluronic acid tablets, D-mannose, palmitoylethanolamide (PEA) local gel. In 2022, cystoscopy meatus was put in place, and the results were normal. An area of vaginal metaplasia of the trigone was observed, along with bladder mucosa with normal appearance, the absence of erosive or ulcerative (Hunner) lesions, and no intravesical neoformations. Normally represented vascular network was observed. No glomerulations were observed with gradual bladder detension. Oral therapy consisted of amitriptyline, PEA tablets, arnica cream locally to the vaginal entry in the evening. As needed, EMLA cream was applied in the clitoral area.

Some characteristics of this clinical case and of interstitial cystitis are the remission of symptoms during pregnancy, which occur in the majority of cases. Furthermore, the trend of symptoms over the years has been fluctuating. This is also a fact that marks the trend of most pain syndromes, and of Bladder Pain Syndrome/Interstitial Cystitis. Fluctuation of hormonal and specifically estrogen levels could be a possible explanation.

### 3.2. Histological Analysis

**Figure 1:**
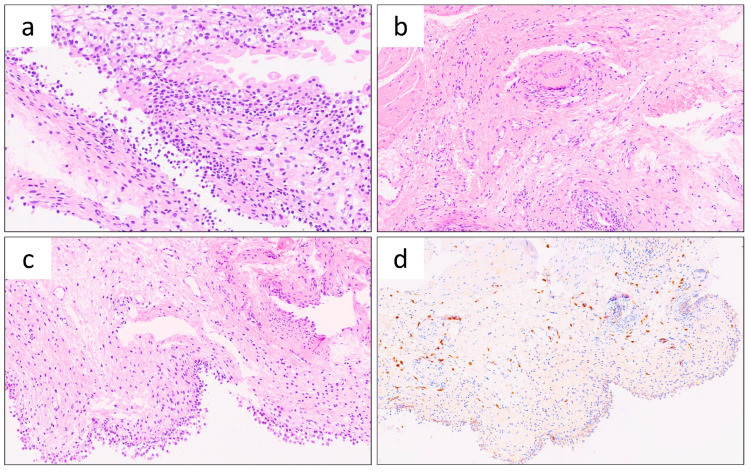
Histological features of the bladder tissue. Tissue sections of bladder were analyzed by hematoxylin-eosin (H&E) stain or by immunodetection. (**a**) Urothelial detachment or denudation (H&E stain). (**b**) Neo angiogenesis and fibrosis of the lamina propria (H&E stain). (**c**) Localized inflammatory process in the lamina propria (H&E stain). (**d**) CD117 immunohistochemistry with mast cells marked in brown. (**a**–**c**): tissue sections stained with hematoxylin-eosin (**d**) CD117 immunohistochemistry: this staining revealed that a major component of this infiltrate consists of mast cells, marked in brown.

Tissue micrographs stained with hematoxylin-eosin (H&E) reveal several key histopathological features indicative of IC (Figure 1).

Urothelial detachment or denudation is caused by a massive subepithelial lymphocytic infiltrate, signifying chronic inflammation. Neoangiogenesis and fibrosis of the lamina propria result from continuous inflammation, characteristic of IC. These changes, including a localized inflammatory process in the lamina propria leading to urothelial denudation, are consistent with IC, a chronic bladder condition characterized by pelvic pain, urgency, and urinary frequency without an apparent bacterial infection. CD117 immunohistochemistry shows that a major component of the inflammatory infiltrate consists of mast cells, marked in brown, serving as significant diagnostic markers for IC. Chronic ulceration and the presence of granulation tissue are associated with Hunner’s lesions, a hallmark of a specific type, less common and clinically severe form of IC, now being considere different from the more common non-Hunner (NH) IC.

### 3.3. NGS Analysis

**Figure 2:**
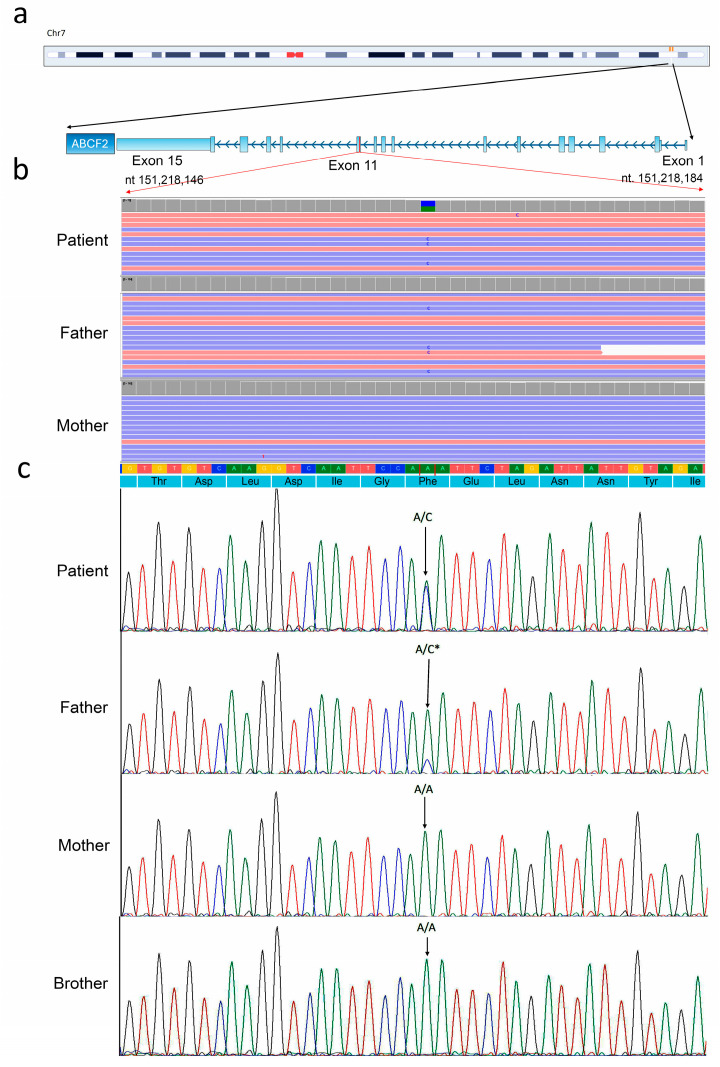
Next generation sequencing (NGS) analysis. (**a**) Graphical depiction of chromosome 7, highlighting the *ABCF2* gene and its exon organization. The red line indicates the position of the *ABCF2* variant on exon 11. This figure was adapted from the UCSC Genome Browser. (**b**) Whole exome sequencing (WES) results for the patient carrying the heterozygous variant c.1253T>G, the father (displaying mosaicism), and the mother. The WES data are presented using the Integrative Genomics Viewer (IGV) visualization tool. (**c**) Conventional Sanger sequencing was performed to confirm the genetic variant identified by WES. This analysis included the affected patient, both healthy parents, and the healthy brother. Notably, the healthy brother did not inherit the genetic variant from the father’s mosaicism. The black asterisk indicates the nucleotide variation observed in the father’s mosaicism.

NGS did not detect variants in known genes associated with the patient’s phenotype. Whole exome sequencing (WES) detected the heterozygous variant c.1253T>G within *ABCF2* gene (NM_007189) in chromosome 7 (Figure 2).

The variant was confirmed by conventional Sanger sequencing and was inherited by the healthy father. The latter, as detected by both WES and Sanger sequencing, displayed a somatic mosaicism of about 20%. This variant was inherited only by the examined patient and not by the healthy brother, as expected. The variant classification according to ACMG guidelines described the variant as likely pathogenic, in alignment with the criteria PM2, PP3, and PP4 (Table 1).

Furthermore, the in silico prediction conducted using various bioinformatic tools consistently classified the variant as pathogenic (Table 2).

Furthermore, data from the global GnomAD database (https://gnomad.broadinstitute.org/) (accessed on 18 December 2024) revealed only one allele (from the African genetic ancestry group) among the total 1,613,786 alleles from the worldwide population, displaying the allele frequency of about 0.00000062. Notably, this specific variant was not found on the European non-Finnish genetic ancestry group (0 alleles among 1,179,724 alleles), which represents the same ethnicity group of the examined individual. PhyloP100way score exhibited a value of 8.637, indicating a high conservation rate of the mutated site across multiple alignment of 99 vertebrate genomes, highlighting its evolutionary significance (Table 3).

**Figure 3:**
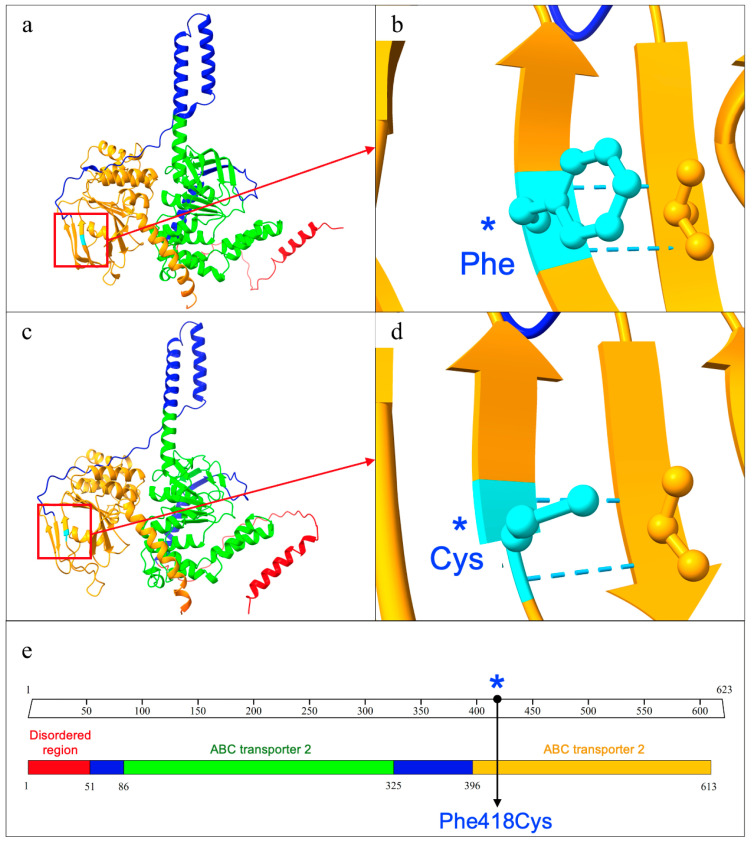
ABCF2 protein structure and domain organization. (**a**) Graphical representation based on the Alphafold prediction of the wild-type ABCF2 protein structure. (**b**) Focus on the wild-type Phenylalanine residue at position 418. (**c**) ABCF2 protein with the mutation Phe418Cys. The different mutated structure in comparison to the wild-type is evident. (**d**) Focus on the mutated Cysteine residue at position 418. (**e**) Functional domains and region organization. The image clearly depicts the mutation site (labelled with the blue asterisk), which is within the ABC transported domain.

The identified mutation is located within the functional domain of ABC transporter 2, spanning from amino acid 396 to the end of the protein at position 613 (Figure 3).

Additionally, the prediction of the ABCF2 protein structure carried out employing UCSF ChimeraX, highlighted differences in hydrogen bond formation between the wild-type and mutated proteins. This variant was a missense mutation that led to the replacement of the amino acid Phenylalanine with Cysteine at position 418 (p.Phe418Cys). Based on the in silico protein structural prediction, the wild-type ABCF2 protein exhibited 687 hydrogen bonds, while the mutated ABCF2 displayed a reduced count of 635 hydrogen bonds. The analysis conducted using the DUET algorithm to assess the mutation’s impact on protein structure revealed a destabilizing effect compared to the wild-type protein. Specifically, the predicted stability change (ΔΔG) value indicated a decrease of −1.781 kcal/mol, signifying an alteration in the energy associated with molecular events.

NGS analysis identified an additional de novo heterozygous variant c.1157 A>C p.Asp386Gly within *INHBB* (NM_002193) gene. Nevertheless, without ruling out the potential contribution of *INHBB* gene in patient’s phenotype, we considered ABCF2 as the putative gene for explaining patient’s symptoms. According to the ACMG criteria, the variant was described as uncertain significance and the in silico prediction by multiple algorithms is reported in Table S1.

## 4. Discussion

Bladder neuropathy, involving nerve damage or dysfunction, is often linked to interstitial cystitis (IC), a chronic condition characterized by pelvic pain and urinary symptoms [3,4,5]. In this study, we report the case of a 33-year-old woman who showed a suspected pudendal nerve neuropathy, not analytically confirmed, and IC, diagnosed by histological analysis. The cystoscopy investigation revealed an increased number of mast cells in the detrusor muscle, indicating chronic inflammation with lymphocytic infiltration (Figure 1). This was confirmed by the presence of Hunner’s lesions. IC/BPS encompasses diverse subtypes, making treatment challenging and necessitating individualized approaches. As documented, neurogenic inflammation involving the pudendal nerve can further amplify the inflammatory processes underlying IC, highlighting its complex interplay with pelvic pain syndromes [6,8]. As previously highlighted, a broad spectrum of neuromodulation approaches has been described for the management of pelvic neuropathies, including IC, pudendal neuralgia and persistent genital arousal disorder [28].

Concerning the examined patient, therapy included Laroxyl 25 mg, palmitoylethanolamide (PEA) twice daily, lipoic acid tablets, hyaluronic acid tablets, D-mannose, and PEA local gel. It is noteworthy that this treatment is not specific for IC/BPS, as there is currently no specific therapy available. However, this treatment aims to replenish the defective glycosaminoglycans (GAG) layer deficiency, and to modulate painful sensation of bladder filling, frequency and urgency. We underscore that, unfortunately, no assessment was performed in the patient examined in this study for analytical determining the pudendal nerve neuropathy.

WES revealed a heterozygous variant within the *ABCF2* gene (Figure 2). To date, this gene has not been assigned a MIM phenotype code number in the OMIM database, associating it with a specific phenotype. Furthermore, according to both the HGMD and LOVD databases, no pathogenic variants have previously been linked to any diseases. The genetic variant identified in the patient was inherited from the healthy father, who exhibits somatic mosaicism at approximately 20%. Mosaic mutations often go undetected, potentially underlying genetic diseases, and may be transmitted to the next generation as pathogenic constitutional variants [29,30]. Notably, this is a pivotal consideration for providing tailored genetic counseling.

WES analysis identified a novel variant in the *INHBB* gene, which encodes the Inhibin Beta B protein, a subunit of inhibin and activin, key regulators of reproductive physiology, including the secretion of follicle-stimulating hormone (FSH) [31]. These proteins play a crucial role in reproductive development and cellular growth. Although *INHBB* is expressed in the granulosa cells of ovarian follicles, we considered *ABCF2* as the primary candidate gene to explain the patient’s symptoms without excluding the potential contribution of *INHBB* or other genetic variants not detected by WES in shaping the complex patient’s phenotype.

The analysis conducted using various in silico algorithms to assess the potential causative effect of the identified variant within ABCF2 gene has classified the mutation as having pathogenic significance (Table 1 and Table 2). The protein structure prediction carried out unveiled notable structural changes within the mutated ABCF2 protein. PhastCons100way and PhyloP100 scores, along with fitCons-gm and Bstatistic scores, collectively suggest that the mutation site exhibits high conservation among species, indicating its potential functional importance across diverse evolutionary lineages (Table 3). This observation implies that the genomic region in question is unlikely subject to background selection, as indicated by the absence of substantial loss of neutral genetic diversity. Based on these findings, which denote the high conservation of this functional region, we hypothesize a plausible causative effect of the identified variant (Table 3). This observation implies that the genomic region in question is unlikely subject to background selection, as indicated by the absence of substantial loss of neutral genetic diversity. Based on these findings, which denote the high conservation of this functional region, we hypothesize a plausible causative effect of the identified variant.

The interplay between ABC transporters, purinergic receptors, and pudendal nerve neuropathy is linked to inflammation and IC [4]. As annotated, ABCF2 enables the ATP-binding (GO:0005524) and ATP hydrolysis (GO:0016887) activities. It is localized into the plasma membrane (GO:0005886) and in the lipid bilayer along with all the proteins and protein complexes (GO:0016020). As extensively reported, there is a well-established correlation between defects in genes involved in ATP metabolism and neuropathy [32]. Furthermore, alterations in ATP metabolism resulting in intracellular ATP accumulation have been identified as a causal factor in neuropathic pain [33]. The identified mutation is located within the functional domain of ABC transporter 2, spanning from amino acid 396 to the end of the protein at position 613 (Figure 3). This domain is involved in the hydrolysis of ATP to energize various biological systems (PRU00434).

Although a specific gene has not yet been identified, many authors have pointed to possible genetic causes related to this condition [11]. Within this context, in agreement with MalaCard database, 29 non-elite and text-mined genes associated with IC as well as about 30 associated super pathways have been identified. Nevertheless, interstitial cystitis (IC) has a prevalence of approximately 0.2% per women in the USA [34]. Additionally, the variant described in the present study displays the frequency of only one allele among the 1,613,786 total alleles.

As highlighted by histological analysis, the patient was diagnosed with interstitial cystitis, accompanied by an increase in mast cells, which, as indicated by several studies, signify an ongoing inflammatory process due to developed neuropathy [35]. In this context, we do not exclude that the inflammation may have been caused by dysregulation of the ABCF2 gene, which, as mentioned earlier, is involved in the ATP metabolism pathway. In fact, one of the main objectives of this study is to highlight the potential genetic causes of IC/BPS to facilitate the development of targeted therapies for this syndrome.

This study aims to expand knowledge about this rare pathology, laying the groundwork for the development of appropriate pharmacological or even genetic therapies. A study in mice showed that introducing a precursor gene for an opioid peptide into the bladder increased endorphin levels, reducing pain caused by chronic inflammation, suggesting localized opioid expression as a potential bladder pain treatment [36]. We emphasize that for the successful planning and development of targeted gene therapy, both *ABCF2* and *INHBB* genes will be tested. This approach aims to confirm the suspected role of ABCF2 in contributing to the patient’s phenotype, particularly in relation to ATP metabolism. Furthermore, the evaluation of INHBB, given its involvement in reproductive and cellular growth processes, is crucial to exclude or confirm its potential contribution to the patient’s clinical presentation. We aim to pinpoint the underlying genetic drivers, which will provide essential insights for designing precise therapeutic interventions.

Despite the precise role of ABCF2 being unclear, we hypothesize that its disruption could lead to defects in ATP metabolism, particularly affecting ATP-binding and hydrolysis processes. In fact, we posit that the disruption of ABCF2 may influence the nociceptive response, highlighting the plausible association between this genetic alteration IC. As recently studied, the modulation of ATP metabolism could represent a promising avenue for therapeutic intervention in managing neuropathic pain [37]. We sincerely hope that our study serves as a foundation for further research aimed at identifying a specific therapy for this rare disease.

## 5. Conclusions

IC/BPS is a rare chronic bladder disorder of unknown cause, characterized by pain, discomfort, or pressure in the bladder, often with frequent and urgent urination. Symptoms range from mild to severe, with various subtypes having different causes and presentations. This variability makes treatment challenging and necessitates a tailored approach for each patient. In the present study, the patient that we examined showed IC/BPS with Hunner’s lesions, as confirmed by the histological analysis. WES analysis identified a genetic variant, classified as likely pathogenic, within ABCF2, a gene implicated in ATP metabolism. This work aimed to establish a possible association between this gene and IC/BPS, laying the fertile groundwork for a possible future development of a genetic therapy targeting the dysregulation involved in this condition.

## Figures and Tables

**Table 1:** Variant classification according to the ACMG criteria.

Criteria for Classifying Variants	Category Code	Description
Moderate	PM2	Absent from controls (or at extremely low frequency if recessive) in Exome Sequencing Project, 1000 Genomes Project, or Exome Aggregation Consortium
Supporting	PP3	In silico evidence
Supporting	PP4	Patient’s phenotype or family history is highly specific for a disease with a single genetic etiology
ACMG variant classification		Likely pathogenic

**Table 2:** Multiple in silico prediction employing several bioinformatic tools.

Tool	Prediction	Score
BayesDel addAF	Pathogenic Moderate	0.3625
BayesDel noAF	Pathogenic Moderate	0.4645
MetaRNN	Pathogenic Moderate	0.8807
REVEL	Pathogenic Moderate	0.922
MetaSVM	Pathogenic Supporting	0.7992
MetaLR	Damaging	0.8249
CADD	Likely Deleterious	25.2
MVP	Pathogenic Moderate	0.9797
PrimateAI	Pathogenic Moderate	0.8955
FATHMM-MKL	Pathogenic Supporting	0.993
FATHMM-XF	Pathogenic Supporting	0.8988
LIST-S2	Pathogenic Supporting	0.973
LRT	Pathogenic Supporting	0
M-CAP	Pathogenic Supporting	0.3338
MutPred2	Pathogenic Moderate	0.945
PROVEAN	Pathogenic Supporting	−5.44
Polyphen2	Possibly Damaging	0.566
BLOSUM	Non-conservative	−4
DANN	Damaging	0.9866
DEOGEN2	Deleterious	0.7608
FATHMM	Damaging	−3.51
Mutation assessor	Damaging	2.15
MutationTaster	Disease causing	1
SIFT	Deleterious	0.009

**Table 3:** Conservation rate of the specific mutation site.

Conservation Parameter *	Score
PhastCons100way	1.000
PhyloP100way	8.637
fitCons-gm	0.725
Bselection	912

* A detailed description of the conservation parameters analyzed is reported in the Section 2.

## Data Availability

The original contributions presented in this study are included in the article/Supplementary Materials. Further inquiries can be directed to the corresponding author.

## Supplementary Materials

The following supporting information can be downloaded at: https://www.mdpi.com/article/10.3390/genes16030281/s1, Table S1: Multiple in-silico analysis related to the pathogenic prediction of the variant c.1157 A>C p.Asp386Gly within *INHBB* (NM_002193) gene.

## Abbreviations

The following abbreviations are used in this manuscript:

ABCF2	ATP-binding cassette sub-family F member 2
ACMG	American College of Medical Genetics
ATP	Adenosine triphosphate
BPS	Bladder pain syndrome
FSH	Follicle-stimulating hormone
GAG	Glycosaminoglycans
GTEx	Genotype-Tissue Expression
GO	Gene ontology
HGMD	Human Gene Mutation Database
H&E	Hematoxylin-eosin
IC	Interstitial cystitis
IGV	Integrative Genomics Viewer
MAF	Minor allele frequency
NGS	Next generation sequencing
NH	Non-Hunner
PEA	Palmitoylethanolamide
TMD	Transmembrane domain
USA	United States of America
UTIs	Urinary trait infections
WES	Whole exome sequencing
