# Supplementary material for: Potential Role of ABCF2 Gene in Pudendal Nerve Neuropathy and Interstitial Cystitis
Source: Genes (Basel). 2025 Feb 26;16(3):281. doi: 10.3390/genes16030281 (PMC11942409; doi:10.3390/genes16030281)
Supplement: Supplementary file 1 [file genes-16-00281-s001.zip › genes-3470707-supplementary.pdf]

Table S1. Multiple in-silico analysis related to the pathogenic prediction of the variant c.1157 A>C p.Asp386Gly within *INHBB* (NM_002193) gene.

Tool	Prediction	Score
MetaRNN	Pathogenic Strong	0.9825
BayesDel addAF	Pathogenic Moderate	0.3894
BayesDel noAF	Pathogenic Moderate	0.3215
REVEL	Pathogenic Moderate	0.917
MetaLR	Uncertain	0.7681
MetaSVM	Uncertain	0.7138
AlphaMissense	Pathogenic Strong	0.9971
DEOGEN2	Pathogenic Strong	0.9744
MutPred	Pathogenic Strong	0.894
CADD	Pathogenic Moderate	31
EIGEN	Pathogenic Supporting	0.7616
EIGEN PC	Pathogenic Supporting	0.6698
LRT	Pathogenic Supporting	0
M-CAP	Pathogenic Supporting	0.5501
Mutation assessor	Pathogenic Supporting	3.17
PrimateAI	Pathogenic Supporting	0.8404
PROVEAN	Pathogenic Supporting	-5.79
SIFT	Pathogenic Supporting	0.001
BLOSUM	Uncertain	-5
DANN	Uncertain	0.9944
FATHMM	Uncertain	-1.94
FATHMM-MKL	Uncertain	0.9593
FATHMM-XF	Uncertain	0.863
LIST-S2	Uncertain	0.9504
MutationTaster	Uncertain	0.9999
MVP	Uncertain	0.9169
SIFT4G	Uncertain	0.003
